# Co-expressed immune and metabolic genes in visceral and subcutaneous adipose tissue from severely obese individuals are associated with plasma HDL and glucose levels: a microarray study

**DOI:** 10.1186/1755-8794-3-34

**Published:** 2010-08-05

**Authors:** Marcel GM Wolfs, Sander S Rensen, Elinda J Bruin-Van Dijk, Froukje J Verdam, Jan-Willem Greve, Bahram Sanjabi, Marcel Bruinenberg, Cisca Wijmenga, Timon W van Haeften, Wim A Buurman, Lude Franke, Marten H Hofker

**Affiliations:** 1 Department of Pathology and Medical Biology, Medical Biology Section, Molecular Genetics, University Medical Center Groningen, University of Groningen, PO Box 30001, 9700 RB Groningen, the Netherlands; 2 NUTRIM School for Nutrition, Toxicology and Metabolism, Department of General Surgery, Maastricht University Medical Center, Maastricht, the Netherlands; 3 Department of Genetics, University Medical Center Groningen, University of Groningen, PO Box 30001, 9700 RB Groningen, the Netherlands; 4 Department of Internal Medicine, University Medical Center Utrecht, PO Box 85500, 3508 GA Utrecht, the Netherlands; 5 Institute of Cell and Molecular Science, Barts and The London School of Medicine and Dentistry, London E1 2AT, UK

## Abstract

**Background:**

Excessive accumulation of body fat, in particular in the visceral fat depot, is a major risk factor to develop a variety of diseases such as type 2 diabetes. The mechanisms underlying the increased risk of obese individuals to develop co-morbid diseases are largely unclear.

We aimed to identify genes expressed in subcutaneous adipose tissue (SAT) and visceral adipose tissue (VAT) that are related to blood parameters involved in obesity co-morbidity, such as plasma lipid and glucose levels, and to compare gene expression between the fat depots.

**Methods:**

Whole-transcriptome SAT and VAT gene expression levels were determined in 75 individuals with a BMI >35 kg/m^2^. Modules of co-expressed genes likely to be functionally related were identified and correlated with BMI, plasma levels of glucose, insulin, HbA_1c_, triglycerides, non-esterified fatty acids, ALAT, ASAT, C-reactive protein, and LDL- and HDL cholesterol.

**Results:**

Of the approximately 70 modules identified in SAT and VAT, three SAT modules were inversely associated with plasma HDL-cholesterol levels, and a fourth module was inversely associated with both plasma glucose and plasma triglyceride levels (p < 5.33 × 10^-5^). These modules were markedly enriched in immune and metabolic genes. In VAT, one module was associated with both BMI and insulin, and another with plasma glucose (p < 4.64 × 10^-5^). This module was also enriched in inflammatory genes and showed a marked overlap in gene content with the SAT modules related to HDL. Several genes differentially expressed in SAT and VAT were identified.

**Conclusions:**

In obese subjects, groups of co-expressed genes were identified that correlated with lipid and glucose metabolism parameters; they were enriched with immune genes. A number of genes were identified of which the expression in SAT correlated with plasma HDL cholesterol, while their expression in VAT correlated with plasma glucose. This underlines both the singular importance of these genes for lipid and glucose metabolism and the specific roles of these two fat depots in this respect.

## Background

It has been proposed that obesity-induced chronic inflammation in adipose tissue precedes the development of insulin resistance and type 2 diabetes. Many inflammatory mediators have been found to be present at increased levels in obese subjects, including Tumor Necrosis Factor (TNF), C-reactive protein (CRP), interleukin-6 (IL-6), and the neutrophil products myeloperoxidase and calprotectin [1-4]. It was also shown that chronic inflammation in obesity is associated with the influx of macrophages into visceral adipose tissue [5-8]. Visceral adipose tissue (VAT) appears to have a larger effect on metabolism than subcutaneous fat (SAT). For example, individuals with a larger visceral fat mass show increased triglyceride levels and an increased risk of developing obesity co-morbidities such as type 2 diabetes and atherosclerosis. Evidence for this has been found by epidemiological studies relating waist-to-hip ratio or waist circumference with obesity-related co-morbidity [1,9,10]. However, the biological processes that underlie this differential impact of the two fat depots on metabolic disease are still obscure.

Although genome-wide association studies have identified many obesity and type 2 diabetes susceptibility genes, most of the individual differences in disease susceptibility among obese subjects are still unclear. A second hypothesis-free and potentially powerful approach to investigate biological processes in obese individuals is genome-wide expression profiling. The potential of this method is underscored by recent studies that have identified numerous genes differentially expressed after weight loss [11-13]. These genes are candidates to play a role in obesity-related co-morbidities, since weight loss improves the metabolic and inflammatory parameters associated with obesity co-morbidities [14,15]. However, to our knowledge, no studies have reported direct investigation of relationships between obesity-related metabolic traits and genome-wide expression levels in both subcutaneous and visceral adipose tissue within obese individuals.

We determined genome-wide transcription levels in both subcutaneous adipose tissue and visceral adipose tissue obtained from a large group of severely obese patients some of whom had type 2 diabetes and/or non-alcoholic steatohepatitis (NASH). From these data we extracted groups of highly co-expressed genes. Subsequent correlation of these genes with metabolic parameters such as plasma glucose, insulin, cholesterol, triglycerides, and non-esterified free fatty acids revealed genes expressed in adipose tissue that are related to these parameters.

## Methods

### Study population

From April 2006 to January 2009, we recruited 75 severely obese subjects with a BMI between 35 and 70 who underwent elective bariatric surgery at the Department of General Surgery, Maastricht University Medical Centre (Maastricht, the Netherlands). Patients with acute or chronic inflammatory diseases (e.g. auto-immune diseases), degenerative diseases, reported alcohol consumption (>10 g/day), or who used anti-inflammatory drugs were excluded. This study was approved by the Medical Ethics Board of Maastricht University Medical Centre, in line with the ethical guidelines of the 1975 Declaration of Helsinki. Informed consent was obtained in writing from each individual.

### Tissue sampling and RNA isolation

Venous blood samples were obtained after 8 hours fasting on the morning of surgery. All blood samples were collected in pre-chilled tubes and processed for analysis of various metabolic traits (shown in table 1) by routine clinical chemistry. Wedge biopsies of visceral adipose tissue (omentum majus), and subcutaneous adipose tissue (abdominal) were taken during surgery. Type 2 diabetes was defined according to the WHO criteria and NASH was diagnosed according to Brunt's criteria [16]. RNA was isolated using the Qiagen Lipid Tissue Mini Kit (Qiagen, Hilden, Germany, 74804) and RNA quality and concentration was assessed with an Agilent Bioanalyzer (Agilent Technologies, Waldbronn, Germany, 5067-1521).

### RNA pre-hybridization processing and hybridization

Starting with 200 ng of RNA, the Ambion Illumina TotalPrep Amplification Kit was used for anti-sense RNA synthesis, amplification, and purification, according to the manufacturer's protocol (Applied Biosystems/Ambion, Austin, TX, USA). 750 ng of complementary RNA was hybridized to Illumina HumanHT12 BeadChips (Illumina, San Diego, CA, USA) and scanned on the Illumina BeadArray Reader. These micro arrays contain 48,813 different probes targeting 37,812 different genes; some genes are targeted by more than one probe.

### Data normalization and quality control

Data were quantile-quantile normalized per tissue using Genespring GX software (Agilent technologies). Only samples were included that passed quality control filtering, which was based on the median probe intensity, the correlation with all other samples for the same tissue, general behaviour of known housekeeping genes, and principal component analysis over the samples. All expression data has been made freely available by submission to GEO under GSE22070.

### Whole-transcriptome microarray data analysis

To find direct associations between gene expression levels and patient characteristics, Spearman rank correlation coefficients were determined between all available quantile-quantile normalized probe expression values and values of the measured traits. To identify differentially expressed genes in SAT and VAT a Wilcoxon Mann-Whitney U-test was used (as implemented in Genespring GX, Agilent technologies).

Next, for SAT and VAT separately, modules of highly co-expressed genes were constructed using pair wise average-linkage cluster analysis as described earlier [17]. First, Pearson correlation coefficients were determined between all the probes on the microarray. Probes with low expression values were not excluded because it is hard to determine a justified cut-off for exclusion of such probes. In addition, noise signals can be considered to be random and are thus not expected to show any co-expression across patients. We used Pearson correlation coefficients because we applied quantile-quantile normalization to the data and using these coefficients is a generally accepted method to construct co-expression networks. We did not take into account negative correlations between probes because this could lead to clustering of genes that are involved in mutually exclusive processes. After determination of correlation correlations between all possible probe pairs, the strongest correlated probe-pair was selected, and grouped together in a module that was assigned the average expression value of the two probes that constitute this module. After addition of this newly created module to the dataset, the two individual probes were removed from the data and the strongest correlation (either between probe-pairs, module-pairs or probe-module-pairs) in the dataset was again selected. This resulted in either the expansion of a module already created (when the strongest correlation was between a probe and a module or between two modules) or in the creation of a new module (when the strongest correlation was between two probe-pairs). We kept repeating this as an iterative process until the most significantly correlated pair was r < 0.65. To visualize the correlations between probes within the modules we constructed coloured heatmaps by plotting pair-wise correlation values of expression of all the probes within the modules. To calculate significance of overlap in gene content between modules and between different datasets we performed Fisher's exact tests using: http://research.microsoft.com/en-us/um/redmond/projects/mscompbio/fisherexacttest.

The module expression - reflecting the average expression value of the probes constituting that module - was correlated with metabolic traits using a non-parametric Spearman rank correlation coefficient, which we chose because it is the most conservative method. For those modules that correlated with a trait, additional conditional analyses were performed, taking into account the possible confounders: menopausal status, hormone treatment and treatment for diabetes, hypertension, dyslipidemia, along with all the other traits we measured - thus gender, age, BMI, plasma levels of glucose, insulin, HbA_1c_, triglycerides, non-esterified fatty acids, HDL cholesterol, LDL cholesterol, total cholesterol, CRP, ALAT, and ASAT. To gain insight what these modules represent, the Panther classification system [18,19] was used to find over- or underrepresented biological themes in the different modules. To visualize the relevant modules, graphs were created by connecting those genes within the modules that were strongly co-expressed (r > 0.65). Genes residing in modules specific to VAT or SAT or modules correlated to a metabolic trait were functionally annotated by manually inspecting KEGG pathways [20], and Pubmed and OMIM gene descriptions.

### Quantitative RT-PCR

In order to estimate the technical quality of the micro array data we performed a validation experiment using quantitative RT-PCR (qRT-PCR). By using random stratified selection, as proposed in [21], we picked 10 genes that were upregulated in SAT, and 10 genes that were upregulated in VAT (Additional file 1, Table S1). Random stratified selection implies that all the genes upregulated in one fat depot were sorted on fold change values, and divided in 10 bins with an equal amount of genes. Next, from each bin one gene was randomly selected (Additional file 1, Table S1). We also measured expression levels of a gene that showed no difference in expression between the two fat depots in the micro array data ("house keeping gene" HKG; Additional file 1, Table S1). We used the same mRNA as was used for the micro arrays from 5 individuals. We performed triplicate measurements and we used a standard curve to be able to compare absolute transcript quantities. We calculated the fold change values between SAT and VAT relative to the HKG and compared these with the fold changes values we observed in the micro array experiment. Primers to perform the experiment were designed using http://www.autoprime.de[22] and obtained from Biolegio (Nijmegen, The Netherlands). Primer sequences are shown in Additional file 1, Table S1. To perform the qRT-PCR we used SYBR green (Biorad, Veenendaal, The Netherlands) on a 7900HT Fast Real-Time PCR System (Applied Biosystems, Nieuwerkerk aan de IJssel, The Netherlands).

## Results

### Highly variable metabolic disturbances in severely obese subjects

Among the 75 severely obese subjects studied, 25 were suffering from type 2 diabetes while 41 were diagnosed with non-alcoholic steatohepatitis (NASH) (table 1). Plasma glucose levels varied between 4.3 and 14.5 mmol/l and plasma HDL levels ranged from 0.5 and 2.8 mmol/l. As expected, several of the traits were found to be highly correlated. (e.g. glucose, triglycerides, and HbA_1C _levels) (Additional file 2, Table S2). The degree of obesity, as reflected by BMI, was correlated to most of the blood parameters determined, in particular with HDL and insulin.

**Table 1:** Clinical and plasma parameters of the study population

	All individuals		Individuals without type 2 diabetes and NASH		Type 2 diabetes patients		NASH patients		Individuals with type 2 diabetes and NASH	
	No. of individuals/Mean (SD)	Minimal/maximal values	No. of individuals/Mean (SD)	Minimal/maximal values	No. of individuals/Mean (SD)	Minimal/maximal values	No. of individuals/Mean (SD)	Minimal/maximal values	No. of individuals/Mean (SD)	Minimal/maximal values
Number of individuals	75		29		25		41		20	
Male/female	22/53		7/22		7/18		12/29		4/16	
Age (years)	44.4 (9.9)	17 - 67	40.3 (9.2)	17 - 54	48.2 (9.4)	28 - 67	45.8 (8.9)	28 - 65	46.5 (8.1)	28 - 65
BMI (kg/m^2^)	46.5 (9.6)	34.6 - 73.6	42.6 (6.5)	34.6 - 56.8	49.4 (11.4)	34.8 - 73.6	48.7 (10.8)	34.8 - 73.6	49.6 (12.1)	34.8 - 73.6
Glucose (mmol/l)	6.60 (2.1)	4.3 - 14.5	5.53 (0.49)	4.3 - 6.5	8.73 (2.5)	4.3 - 14.5	7.16 (2.56)	4.3 - 14.5	8.8 (2.72)	4.3 - 14.5
Insulin (mU/l)	19.3 (10.9)	3.8 - 53	16.7 (8.8)	5.6 - 37	20.6 (13.1)	5.5 - 53	20.9 (11.1)	3.8 - 49	20.4 (10.9)	3.8 - 49
HbA_1c_	6.58 (1.39)	5.1 - 12.1	6 (0.54)	5.1 - 7.2	7.9 (1.78)	5.3 - 12.1	6.88 (1.65)	5.2 - 12.1	7.85 (1.88)	5.2 - 12.1
Total cholesterol (mmol/l)	5.08 (1.1)	3 - 9.8	4.93 (0.65)	3.5 - 6.1	5.15 (1.43)	3.5 - 9.8	5.08 (1.23)	3 - 9.8	5.2 (1.5)	3 - 9.8
HDL cholesterol (mmol/l)	0.98 (0.38)	0.5 - 2.8	1.05 (0.3)	0.6 - 1.9	0.8 (0.23)	0.5 - 1.3	0.93 (0.44)	0.5 - 2.8	0.78 (0.22)	0.5 - 2.8
LDL cholesterol (mmol/l)	3.22 (1.02)	1.1 - 7.4	3.16 (0.86)	1.1 - 4.3	3.29 (1.18)	1.5 - 7.4	3.17 (1.06)	1.5 - 7.4	3.32 (1.25)	1.5 - 7.4
TG (mmol/l)	2.26 (2.12)	0.63 - 16.4	1.67 (0.95)	0.63 - 4.2	3.31 (3.46)	0.88 - 16.4	2.73 (2.71)	0.88 - 16.4	3.56 (3.67)	0.88 - 16.4
NEFA (mmol/l)	0.64 (0.32)	0.12 - 1.66	0.52 (0.34)	0.12 - 1.66	0.72 (0.3)	0.16 - 1.33	0.71 (0.28)	0.21 - 1.33	0.76 (0.29)	0.21 - 1.33
ALAT (U/l)	25.7 (16.5)	6 - 124	19.8 (6.3)	7 - 37	32.3 (23.1)	10 - 124	27.7 (19.3)	6 - 124	32.4 (24.8)	6 - 124
ASAT (U/l)	25.3 (12.9)	7 - 72	21.1 (11.6)	7 - 50	28.9 (15.4)	8 - 72	28.4 (13)	8 - 72	30.7 (16)	8 - 72
CRP (mg/l)	10.2 (8.5)	1 - 37	9.9 (9.2)	1 - 37	13.1 (10)	1.7 - 35	10 (8.2)	1.7 - 35	12.8 (10.2)	1.7 - 35

Data are means ± standard deviation (SD) and the minimal and maximal values for each trait. Data are given for all individuals, individuals without type 2 diabetes or NASH, type 2 diabetes patients, NASH patients, and individuals with both type 2 diabetes and NASH. Patients with type 2 diabetes are 36, 37, 40, 42, 43, 49, 51, 54, 55, 57, 60, 63, 64, 65, 69, 75, 77, 78, 79, 85, 87, 97, 101, 103, and 110 - IDs are concordant with the IDs used submitted to the Gene Expression Omnibus database. Patients with NASH are 38, 42, 43, 44, 45, 47, 49, 51, 52, 53, 54, 57, 58, 60, 62, 63, 64, 65, 69, 72, 75, 78, 79, 83, 85, 87, 88, 92, 93, 95, 97, 99, 100, 101, 102, 103, 104, 105, 107, 110, and 113. TG, triglycerides; NEFA, non-esterified fatty acid; ALAT, alanine aminotransaminase; ASAT, aspartate aminotransaminase; CRP, C-reactive protein; NASH, non-alcoholic steatohepatitis.

### Identification of genes overexpressed in subcutaneous and visceral adipose tissue

We determined genome-wide gene expression profiles of SAT and VAT to identify the gene expression differences in adipose tissue that could potentially underlie the variation described in metabolic traits. After quality control, 73 SAT samples and 69 VAT samples were retained for further analysis. RNA Integrity Numbers (RIN) of these samples ranged between 6.5 and 8.7 in SAT (average 7.6), and 6.2 and 9.4 in VAT (average 7.5). The qRT-PCR validation experiment showed that the 20 genes we tested showed very similar fold change values (correlation coefficient r = 0.88 between the microarray fold change and qRT-PCR fold change for the 20 genes) in the qRT-PCR and micro array experiments (Additional file 3, Table S3 and Additional file 4, Figure S1).

Preliminary analysis of the gene expression profiles revealed that 1,344 genes were significantly upregulated (p < 0.05 after Bonferroni correction) in SAT compared to VAT, with 609 genes showing a >1.5-fold change. In VAT, we identified 1,246 genes with a significantly higher expression compared to SAT (p < 0.05 after Bonferroni correction). Of these, 909 showed >1.5-fold increase (Additional file 5, Table S4A and S4B).

Remarkably, a large number of genes (138 genes) had a more than ten-fold higher expression in VAT than in SAT, whereas only 20 genes were upregulated by more than ten-fold in SAT (Additional file 5, Table S4A and S4B). Subsequent gene-set enrichment analysis of the 138 genes specifically expressed in VAT using Panther revealed that they are involved in signal transduction, cell adhesion, cell communication, and developmental processes (Bonferroni corrected p < 0.05). The 20 genes that showed >10-fold higher expression in SAT are highly enriched in homeobox transcription factors (*HOXA9*, *HOXA10*, *HOXC8*, *HOXC9*, *IRX2*, and *IRX5*). Additional file 6, Table S5A and S5B, shows the overrepresented biological processes in the groups of genes that are differentially expressed in VAT and SAT with different cut-offs based on fold change (fold change > 1, fold change > 1.5, and fold change > 10). Some biological Panther processes are overrepresented in the lists of genes with a fold change >1.5 in both VAT and SAT (p < 0.05 after Bonferroni correction). This might indicate that these processes - as defined by the Panther classification system - are important in both VAT and SAT, but that these processes are differently moderated in the distinct fat depots. Panther biological processes that are overrepresented amongst the genes upregulated >1.5-fold in both SAT and VAT are "lipid, fatty acid, and steroid metabolism", cell structure and motility, developmental processes, cell adhesion, neurogenesis, ectoderm development, immunity and defense, signal transduction, cell adhesion-mediated signalling, and cell communication. Biological processes specifically present in genes upregulated in SAT are cell structure and vitamin metabolism. In VAT such specific processes are "receptor protein tyrosine kinase pathway", cell surface receptor mediated signal transduction, mesoderm development, ligand-mediated signalling, complement mediated immunity, muscle contraction, cell proliferation and differentiation, extracellular matrix protein-mediated signalling, neuronal activities, cell cycle control, ion transport, protein modification, protein phosphorylation, other developmental process, oncogenesis, cation transport, and transport.

### Gene modules based on co-expression can classify genes reliably

**Figure 1:**
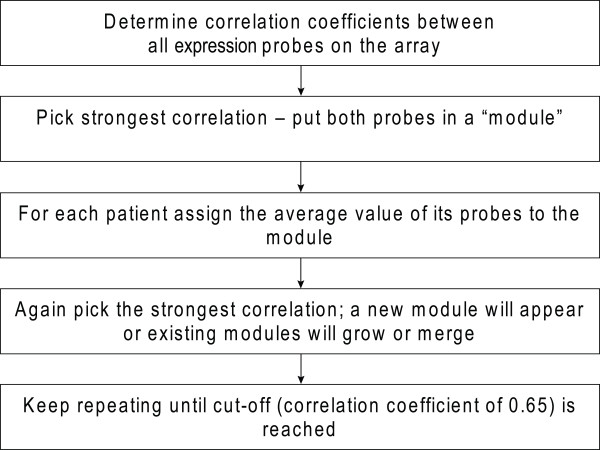
**Bioinformatic data processing showing the steps that were used to generate modules**.

The expression levels of many individual genes showed strong correlations with metabolic traits. For example, as expected BMI and leptin mRNA levels in VAT were highly correlated (r = 0.51; p = 1.1 × 10^-5^). Other examples included expression levels in subcutaneous fat of CD68 molecule (*CD68*) (r= -0.62; p = 2.1 × 10^-8^), CD300a molecule (*CD300A*) (r= -0.58; p = 2.4 × 10^-7^), and sterol regulatory element binding transcription factor 1 (*SREBF1*) (r = 0.53; p = 3.1 × 10^-6^), which were correlated with plasma levels of HDL cholesterol. However, given the scale of the experiment, it was difficult to extract meaningful correlations on a gene-by-gene basis. We therefore chose to apply a clustering method that enabled us to identify sets of functionally related genes linked to the phenotypic traits. For this reason, co-expressed genes were grouped together in "modules" (figure 1).

In SAT, we identified 67 modules containing 5 or more genes. These modules comprised 3,263 genes in total. In VAT, 4,509 genes could be grouped into 76 modules of 5 or more genes (Additional file 7, Table S6A and S6B). Additional files 8 and 9, Figure S2 and Figure S3 respectively, show coloured heat-maps of pair-wise correlations between genes residing in the modules identified in SAT and VAT. Bright red indicates a strong negative co-expression, whereas bright green indicates strong positive co-expression. Expression of genes within a single module are strongly correlated whereas genes that belong to different modules generally do not show strong co-expression. As expected, some genes residing in different modules are strongly negatively correlated to each other, as module construction was solely based on gene-pairs, showing strong positive co-expression.

To confirm that the modules represented coherent biological processes, an analysis was performed using the Panther gene classification tool. Importantly, groups of genes known to be functionally related were indeed overrepresented in most of the modules (Additional file 10, Tables S7A and S7B), indicating that our modules reflect consistent biological mechanisms. In addition, since visceral and subcutaneous adipose tissues are closely related, we expected to find similar modules in both tissues. Indeed, most of the modules detected in one of the adipose tissues had a counterpart containing mainly the same genes in the other adipose tissue. The overlap in gene content between modules in VAT and SAT was confirmed by performing Fisher's exact tests (table 2). This again supports the notion that these modules represent a reliable classification of genes. There was no module present in SAT with similar contents as module VAT 4. This module largely consisted of genes that were higher expressed in VAT than in SAT, and thus likely represents a process predominantly present in VAT. Biological processes overrepresented in this module are similar to those found in genes strongly higher expressed in VAT than SAT (Additional file 6, Table S5B).

**Table 2:** Overlap between genes in the 10 largest modules identified in subcutaneous (SAT) and visceral adipose tissue (VAT)

		SAT 1	SAT 2	SAT 3	SAT 4	SAT 5	SAT 6	SAT 7	SAT 8	SAT 9	SAT 10
	**Total**	**723**	**554**	**344**	**308**	**146**	**75**	**87**	**87**	**103**	**46**

**VAT 1**	**469**	20	1	27	2	*^5 ^69	*^6 ^29	0	0	0	0
**VAT 2**	**641**	*^1 ^536	3	12	1	1	7	0	0	8	1
**VAT 3**	**524**	9	*^2 ^307	1	7	0	0	0	0	31	0
**VAT 4**	**464**	5	2	2	16	5	1	*^7 ^27	3	0	0
**VAT 5**	**306**	1	63	0	*^4 ^56	0	0	0	4	4	0
**VAT 6**	**253**	6	0	*^3 ^115	1	1	0	0	0	0	0
**VAT 7**	**193**	5	0	32	0	6	0	0	0	0	1
**VAT 8**	**209**	27	23	1	0	0	0	0	0	*^9 ^58	0
**VAT 9**	**103**	0	0	0	32	0	0	1	*^8 ^54	0	0
**VAT 10**	**104**	0	60	0	1	0	0	0	0	5	0

After generation of modules in both types of adipose tissue, the overlap in gene content of the 10 largest modules in VAT and SAT was determined. As an example, modules VAT 2 and SAT 1 are largely the same since approximately 75% of the genes present in one of these modules is also present in the other. For most, although not all, of the modules in one adipose tissue there is a similar module in the other adipose tissue. P-values to determine the significance of the overlap between the modules were performed using a Fisher's exact test: *1: p = 1.54 × 10^-321^; *2: p = 1.20 × 10^-321^; *3: p = 2.07 × 10^-138^; *4: p = 1.58 × 10^-43^; *5: p = 5.74 × 10^-73^; *6: p = 6.65 × 10^-28^; *7: p = 3.33 × 10^-23^; *8: p = 2.16 × 10^-107^; *9: p = 1.33 × 10^-89^. Genes within module SAT 10 overlap with genes within module VAT 11 (not shown).

### Modules of co-expressed adipose tissue genes associated with specific metabolic traits

**Figure 2:**
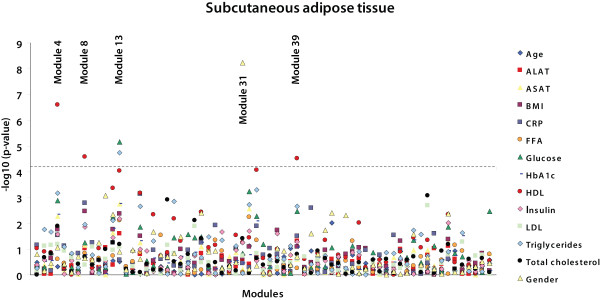
**Correlations between modules in subcutaneous adipose tissue and metabolic traits**. -Log p-values for Spearman rank correlation coefficients between the values of the modules and the different metabolic traits are shown for subcutaneous adipose tissue. Modules are ordered on the X-axis in the same way as in Additional file 7, Table S6A and S6B; thus with the largest module - containing the largest number of probes - on the left. The dashed line represents a highly stringent Bonferroni corrected p-value of 0.05.

**Figure 3:**
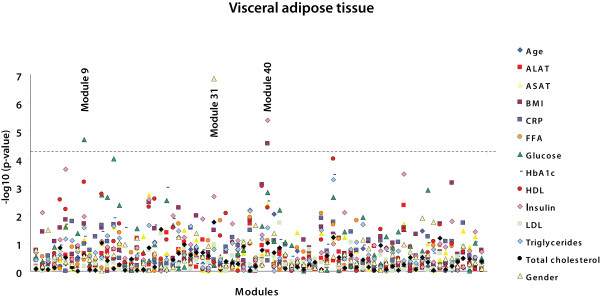
**Correlations between modules in visceral adipose tissue and metabolic traits**. -Log p-values for Spearman rank correlation coefficients between the values of the modules and the different metabolic traits are shown for visceral adipose tissue. Modules are ordered on the X-axis in the same way as in Additional file 7, Tables S6A and S6B; thus with the largest module - containing the largest number of probes - on the left. The dashed line represents a highly stringent Bonferroni corrected p-value of 0.05.

Analyses in which we investigated differences in gene expression between patient groups - i.e. type 2 diabetes and non-alcoholic steatohepatitis - did not yield statistically significant results because our dataset has insufficient power. This is most likely due to complexity of these phenotypes. Therefore the modules were analyzed for correlation with various continuous traits of the obese individuals (figures 2 and 3). In SAT, five modules were significantly associated with a trait after correcting for multiple testing (p < 5.33 × 10^-5^; Bonferroni corrected p < 0.05) (table 3). Three of these modules (SAT 4, SAT 8, and SAT 39) were inversely correlated to plasma HDL-cholesterol levels. One module (SAT 13) showed a correlation to both plasma glucose and plasma triglyceride levels, and another (SAT 31) was correlated to gender. In VAT, three modules were significantly correlated with a trait (p < 4.64 × 10^-5 ^; Bonferroni corrected p < 0.05) (table 3). VAT 9 was correlated to plasma glucose levels, VAT 40 was correlated to both plasma insulin levels and BMI, and VAT 31 was correlated to gender.

**Table 3:** Modules correlated with metabolic traits in subcutaneous (SAT) and visceral adipose tissue (VAT)

Tissue	Module ID	Correlated trait	No. of genes in module	Correlation coefficient	P-value
SAT	31	Gender	14		6.6 × 10^-66^
	4	HDL cholesterol	308	-0.58	2.5 × 10^-7^
	13	Glucose	28	-0.51	7.0 × 10^-6^
		Triglycerides		-0.50	1.8 × 10^-5^
	8	HDL cholesterol	87	-0.49	2.7 × 10^-5^
	39	HDL cholesterol	7	-0.49	3.0 × 10^-5^
					
VAT	31	Gender	14		8.2 × 10^-60^
	40	Insulin	12	-0.54	4.3 × 10^-6^
		BMI		-0.49	2.8 × 10^-5^
	9	Glucose	103	0.50	2.1 × 10^-5^

Only those modules significantly correlated to a metabolic trait after Bonferroni correction are shown, with the names of the modules, the traits to which they were correlated, the number of genes that comprise the module, and the Spearman rank correlation coefficient between the value of the module - i.e. the average of all the probes in the module - and the trait. To calculate p-values for association between a module and gender we used a students T-test.

Correlations between the modules, associated to a trait, and all the traits were recalculated taking into account various potential confounding factors. Such confounding factors might be women's menopausal status, the use of hormone therapy, and treatment for diabetes, hypertension, or dyslipidemia (Additional file 11, Table S8). Age, gender, menopausal status, hormone treatment, and treatment for diabetes, hypertension, and dyslipidemia did not influence the results of the uncorrected correlation analysis (Additional file 12, Table S9A and S9B). Correction for BMI showed that BMI is a confounder for the correlations between plasma insulin levels and module VAT 40, which is in line with the significant correlation between module VAT 40 and both BMI and plasma insulin levels. BMI also confounds the correlation between module SAT 8 and plasma HDL levels. However, since insulin and BMI are not correlated to this module if corrected for plasma HDL levels (p-values of 0.276 and 0.331 respectively) we conclude that plasma HDL levels, and not BMI or plasma insulin levels, drive module SAT 8.

**Figure 4:**
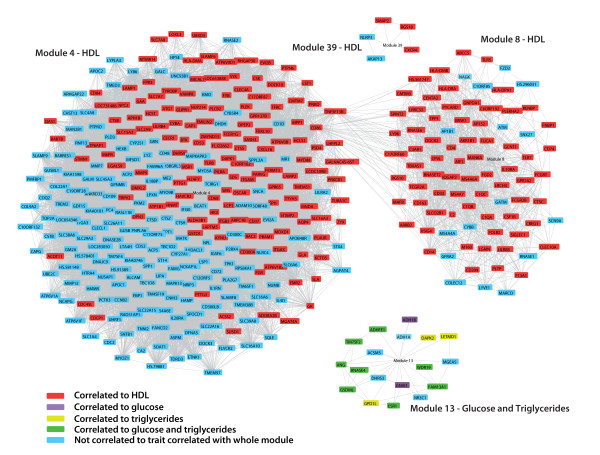
**Gene co-expression network of modules in subcutaneous adipose tissue correlated to a trait**. Genes in a module that have an individual Pearson correlation coefficient >0.65 are connected. Genes that are individually correlated (p < 0.01) to the metabolic trait to which the whole module is correlated are shown in red (HDL), purple (glucose), yellow (triglycerides), or green (both glucose and triglycerides). The *CD86 *and *TNFSF13B *genes are present in both modules SAT 4 and SAT 8 because different probes targeting this gene are highly correlated to different modules.

**Figure 5:**
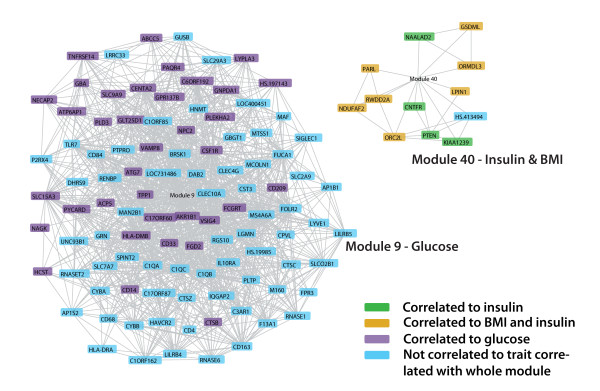
**Gene co-expression network of modules in visceral adipose tissue correlated to a trait**. Genes in a module that have an individual Pearson correlation coefficient >0.65 are connected. Genes that do not have an individual correlation of >0.65 with any other gene in the module are not shown. Genes that are individually correlated (p < 0.01) to the metabolic trait to which the whole module is correlated are shown in purple (glucose), green (insulin), or yellow (BMI and insulin).

Figures 4 and 5 show gene co-expression networks that consist of all the genes that reside in modules associated to a metabolic trait and that are individually strongly correlated - r > 0.65 - to another gene within the module. Some genes that belong to the module are thus not included in these figures because they are not strongly correlated to an individual gene, but instead they are strongly correlated to the average of a set of genes that make up the module. However most of the genes that are present in a module have strongly correlated expression levels to other individual genes within the same module, which validates our approach. Moreover most of the genes within a module are individually correlated - although not significant after stringent correction - to the trait that is correlated with the whole module, which is depicted by different colours in figures 4 and 5.

These data suggest that genes co-expressed in SAT mainly modulate plasma HDL levels, while genes co-expressed in VAT may affect plasma glucose and insulin levels, thereby contributing to the development of type 2 diabetes.

### Genes co-expressed in adipose tissue involved in immune and metabolic processes

To further define the biological mechanisms represented by the genes in the modules correlated to the metabolic traits, we used the Panther gene classification tool (table 4). Module SAT 4, consisting of 308 genes, harboured significantly more genes than would be expected by chance that are involved in immunity and defense, humoral immunity (B-cell, T-cell), cell adhesion, transport, signal transduction, ion transport (cation transport), intracellular signalling, carbohydrate metabolism, and "lipid, fatty acid, and steroid metabolism". SAT 8 contained 87 genes and was markedly enriched in genes involved in immunity and defense, humoral immunity (B-cells, T-cells, MHCII), innate immunity (complement-mediated, macrophages), and endocytosis. VAT 9 harboured 103 genes and correlated with plasma glucose levels. It contained many genes involved in immunity and defense, macrophage-mediated immunity, cell adhesion, and transport. The genes in this module resemble the genes found in SAT modules 4 and 8; 32 genes are present in both SAT 4 and VAT 9, and 54 genes are present in SAT 8 and VAT 9. The other modules correlated to a metabolic trait (SAT 13, SAT 39, VAT 40) but could not be used for reliable pathway analyses since the number of genes residing in these modules is too small.

**Table 4:** Over-represented Panther biological processes in modules correlated to a metabolic trait in subcutaneous (SAT) and visceral adipose tissue (VAT)

	SAT Module 4		SAT Module 8		VAT Module 9	
	**No. of genes in module**	**P-value**	**No. of genes in module**	**P-value**	**No. of genes in module**	**P-value**

Total	308		87		103	
Signal transduction	67	2.8 × 10^-4^	NA	NS	NA	NS
- Intracellular signalling cascade	26	2.0 × 10^-3^	NA	NS	NA	NS
Immunity and defense	43	3.5 × 10^-9^	31	4.1 × 10^-17^	21	8.2 × 10^-7^
- T-cell-mediated immunity	13	9.2 × 10^-5^	7	6.4 × 10^-4^	NA	NS
- MHCII-mediated immunity	NA	NS	5	2.5 × 10^-5^	NA	NS
- Macrophage-mediated immunity	NA	NS	7	7.5 × 10^-5^	5	3.3 × 10^-2^
- Endocytosis	NA	NS	7	6.2 × 10^-3^	NA	NS
- B-cell and antibody-mediated immunity	7	2.4 × 10^-2^	4	4.9 × 10^-2^	NA	NS
- Complement mediated immunity	NA	NS	4	2.4 × 10^-3^	NA	NS
Transport	41	2.8 × 10^-7^	NA	NS	16	1.2 × 10^-3^
- Ion transport	23	1.8 × 10^-4^	NA	NS	NA	NS
Cell adhesion	20	1.6 × 10^-3^	NA	NS	11	9.2 × 10^-4^
Lipid, fatty acid and steroid metabolism	21	9.7 × 10^-3^	NA	NS	NA	NS
Carbohydrate metabolism	19	2.5 × 10^-3^	NA	NS	NA	NS
Other	89	NA	37	NA	44	NA
Unclassified	76	NA	19	NA	24	NA

SAT modules 4 and 8 correlate to HDL, and VAT module 9 correlates to glucose. The modules SAT 12, SAT 39, VAT 40 do not contain enough genes to perform a reliable pathway analysis. The modules correlated to gender in both tissues consist solely of Y-chromosomal genes and are not included in the table.

Only biological functions that are over-represented in a module after Bonferroni correction are shown. For each over-represented biological function the number of genes in the module that belong to this biological function, and the corresponding Bonferroni corrected p-value for over-representation are shown. The number of genes that are part of a class which is not over- or under-represented, and the number of genes that could not be classified by PANTHER are also shown for each module ("other" and "unclassified" respectively). NA, not applicable; NS, not significant.

**Figure 6:**
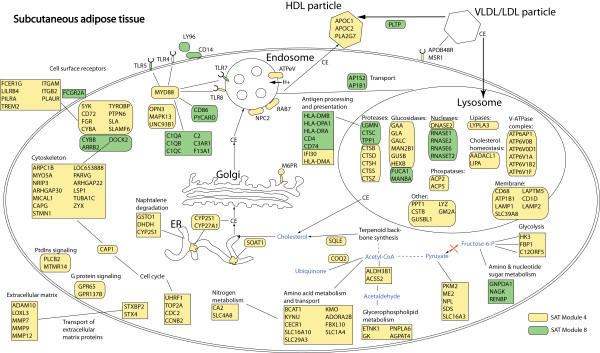
**Overview of pathways in subcutaneous adipose tissue related to plasma HDL-cholesterol levels**. KEGG pathways, and PubMed and OMIM gene descriptions were used to generate an overview of the functions of the genes in SAT modules 4 and 8. Of the 395 genes present in both modules, approximately 200 genes could be assigned a function related to other genes in the two modules. These approximately 200 genes were included in the figure in order to present a schematic overview of processes involved in SAT modules 4 and 8. There is evidence that modules SAT 4 and SAT 8 represent different biological processes, but it is unclear what these differences exactly are. Genes in yellow belong to module SAT 4 and genes in green belong to SAT 8. Cellular trafficking of cholesterol is indicated with arrows. CE, cholesteryl esters.

To further explore the functional relationships between genes in SAT modules 4 and 8 we used KEGG pathway analysis, and PubMed and OMIM gene descriptions. We did not focus on module VAT 9 because this module contains the same but less genes as modules SAT 4 and SAT 8 and is therefore less preferable for pathway analyses. Figure 6 shows an overview of genes that are present in SAT modules 4 and 8, and that, based on current knowledge, could be assigned a certain biological function. Interestingly, SAT 4 genes (classified by Panther analysis to be involved in "carbohydrate metabolism", and "lipid, fatty acid, and steroid metabolism") encode proteins involved in the structure or modification of the HDL particle, lysosomal degradation, and cholesterol metabolism and trafficking in macrophages. Furthermore, the presence of certain metabolic genes points towards a decrease of glycolysis, increased generation of pyruvate, acetate, and acetaldehyde from other sources than glycolysis, and upregulation of amino acid metabolism and glycerophospholipid metabolism.

Among those genes residing in both SAT 4 and SAT 8 involved in immune-related processes, several are members of well-described pathways. As an example, several members of Fcγ receptor-mediated signalling are present in SAT 4 and 8, including *FCGR2A*, *FCGR2B*, Gardner-Rasheed feline sarcoma viral (v-fgr) oncogene homolog (*FGR*) [23,24], spleen tyrosine kinase (*SYK*) [25,26], protein tyrosine phosphatase 6, non-receptor type 6 (*PTPN6*; also called *SHP-1*), TYRO protein tyrosine kinase binding protein (*TYROBP*; also called *DAP12*), and cytochrome b-245, alpha polypeptide (*CYBA*) [27,28]. Another example of a pathway that is overrepresented amongst the genes present in modules SAT 4 and SAT 8 is the Toll-like receptor signalling pathway. Genes that belong to this pathway are Toll-like receptor 5, 7, and 8 (*TLR5*, *TLR7*, *TLR8*), CD14 molecule (*CD14*), lymphocyte antigen 96 (*LY96*; also known as *MD-2*), Bruton agammaglobulinemia tyrosine kinase (*BTK*) [29], and myeloid differentiation primary response gene (88) (*Myd88*). Our data thus suggest that some genes in SAT 4 and SAT 8 are involved in immune-related signalling pathways such as the Toll-like receptor signalling and the Fcγ receptor-mediated signalling pathways.

## Discussion

In this study, we have identified genes expressed in SAT and VAT that are related to lipid and glucose metabolism parameters in obesity. In particular, plasma levels of HDL cholesterol and glucose were found to be correlated to sets of co-expressed, and thus functionally related, genes (modules). Remarkably, several SAT modules were correlated to plasma HDL cholesterol levels and one VAT module was correlated to plasma glucose levels, although these SAT modules contained primarily the same genes as the VAT module. This difference highlights the fact that SAT and VAT have a distinct biological role. In silico classification of the co-expressed genes revealed that a significant number are involved in immunity and metabolism. This is in line with the concept that the immune and metabolic systems are tightly interconnected and that this interconnection is pivotal in the development of co-morbidities of obesity.

Several of the genes we have identified in this study play a role in pathways or processes that have already been linked to obesity co-morbidity, in particular HDL levels. These pathways or processes include immunity-related signalling pathways, the complement cascade, cholesterol metabolism and trafficking, lysosomal degradation and trafficking, and composition of the HDL particle [30-40]. A crucial finding of this study is the identification of novel genes that are correlated to HDL and glucose levels in severely obese individuals. The role of these genes in obesity co-morbidity is largely unknown, and further research is required to unravel the relationship between these genes and HDL and glucose levels. Possibly these genes may control plasma HDL cholesterol and glucose levels, or they might be involved in the response of adipose tissue to changed plasma HDL and glucose levels.

An earlier micro array study performed by Tchkonia *et al *[41] investigated differences in gene expression levels between differentiated and undifferentiated adipocytes derived from subcutaneous, visceral, and mesenteric adipose tissue. We observed an overlap between the results of this study and our own data that was higher than expected: In our study we identified 1344 genes to be upregulated in SAT and 1246 in VAT. Of these 1344 and 1246 genes, 103 and 87 respectively had also been identified in the study of Tchkonia *et al*, which identified 920 transcripts to be differently expressed across fat depots in either differentiated or undifferentiated cells (overrepresentation p-values: 2.5 × 10^-7 ^for SAT and 8.7 × 10^-5 ^for VAT, Fisher's exact test assuming that 20,000 unique genes were tested in total). Of the 87 genes differentially expressed in the study of Tchkonia *et al *that overlapped with genes upregulated in VAT in our study, 76 (87%) were differentially expressed in undifferentiated adipocytes that had been derived from distinct fat depots. 39 of these 76 genes (51%) were present in module VAT 4, which is substantially higher than expected (p-value: 2.5 × 10^-11^; Fisher's exact test, again assuming that 20,000 different genes were tested). These observations make it tempting to speculate that this module is related to processes in VAT-specific undifferentiated adipocytes. This is line with absence of a module in SAT that contains the same genes as module VAT 4.

Previous studies on the effects of obesity on genome-wide expression levels in SAT revealed several classes of genes to be regulated by obesity [12,13]. Downregulated genes in obesity include lipolytic genes. Upregulated genes include genes controlling the structure and turnover of the extracellular matrix (ECM) and genes of infiltrating immune cells encoding cytokines and plasma membrane proteins. Another study investigating gene expression levels in whole SAT before and after weight loss [11] found similar sets of genes as found in the studies mentioned above [12,13]. A subset of these genes was shown to be linked to glucose disposal rate, indicating that they may be involved in insulin resistance. Among the genes involved in immunity and the ECM, there was an overrepresentation of genes expressed in immune cells (e.g. macrophages), whereas genes involved in lipid metabolism were mostly genes expressed in adipocytes. Investigation of 31 genes specifically expressed in macrophages but not in adipocytes [11] revealed that these genes show significantly different gene expression profiles during weight loss induced by a stringent diet. 2 genes did not respond to this diet, whereas 7 genes responded strongly, 11 genes responded weakly, and another 11 genes showed an intermediate response [11].

In our studies of a group of 75 severely obese individuals, the genes in SAT modules 4 and 8, and VAT module 9 showed significant overlap with the genes differentially expressed after weight loss [11], as well as with the genes differentially expressed between lean and obese individuals [12] (Additional file 13, Table S10A). In addition, genes in these modules overlap with macrophage genes differentially expressed during dietary intervention and with genes predictive of insulin sensitivity (Additional file 13, Table S10B and S10C). Strikingly, of the 31 genes specifically expressed in macrophages but not in adipocytes investigated by Capel et al. [11], 26 are present in SAT modules 4 and 8, which are correlated to HDL levels (p = 2.1 × 10^-40^; Fisher's exact test assuming that 20,000 different genes were tested). Moreover the grouping of these macrophage genes based on different expression patterns during dietary intervention closely resembles the grouping of the genes in SAT modules 4 and 8 generated in our study; 6 of 7 genes identified as high responders to energy restriction regarding their expression are present in module SAT 4, and 10 of 11 genes identified as low responders are present in module SAT 8 [11]. The overlap of genes found in these studies with different designs - comparing lean with obese individuals, studying the same individuals after weight loss, and studying quantitative metabolic traits in obese individuals - supports these approaches and strongly suggests that the genes identified are involved in obesity-related disease mechanisms.

It should be noted that the correlations between the modules and the metabolic traits identified in our study are not driven by BMI, since BMI itself was not correlated to the modules - except VAT 40. The correlation between module SAT 8 and plasma HDL levels was confounded by BMI and plasma insulin levels, but the absence of any correlation between this module and BMI or plasma insulin levels after correction for plasma HDL levels, indicates that HDL is the driver of this module. It can be speculated that module SAT 8 represents a BMI/plasma insulin driven effect of HDL whereas module SAT 4 represents an effect of HDL independent of BMI/plasma insulin.

A remaining question is what biological phenomena are driving the modules correlated to a metabolic trait. Here, we will mainly focus on SAT modules 4 and 8, because these two modules contain the largest number of genes, making it more valid to identify over-represented pathways in them. Capel et al. [11] investigated 31 genes specifically expressed in macrophages but not in adipocytes. Of these, 26 are present in SAT modules 4 and 8, which are correlated to HDL levels. The presence of genes within these modules that are specifically expressed in macrophages, might be a reflection of the relative number of macrophages in the whole adipose tissue and it is possible that SAT modules 4 and 8 are, at least in part, driven by the degree of macrophage infiltration. The presence of two different modules of macrophage genes, as confirmed by grouping of the macrophage genes by Capel et al., might be driven by differences between or within macrophages (e.g. macrophage infiltration or activation). Another possible biological mechanism that might underlie the appearance of SAT modules 4 and 8 are differences in adipocyte size, since with equal numbers of macrophages per m^3 ^the relative amount of macrophage mRNA would increase if adipocytes get larger. Other mechanisms that may operate are the induction of adipocyte autophagy, ER-stress, or inflammasome activation. In order to get insight into these questions histology experiments are required to quantitate macrophage infiltration, adipocyte size, markers of autophagy, ER-stress, and inflammasome activation. Of note, 13 of the 31 macrophage-specific genes were also present in VAT module 9. This overlap is still highly significant, although less striking than in SAT (p = 1.6 × 10^-22^; Fisher's exact test assuming that 20,000 different genes were tested).

Importantly, VAT is the most metabolically active fat depot, and it has been proposed that complications of obesity correlate to an excess of visceral fat rather than to subcutaneous fat accumulation [9,10]. However, many studies investigating gene expression in adipose tissue have only focused on SAT. In our study, we included samples from both fat depots and we identified numerous genes differentially expressed in VAT and SAT. In this regard our results are in line with a previous study in ten nondiabetic, normolipidemic obese men [42], but due to our larger sample size we were able to detect more genes differentially expressed in SAT and VAT.

Unexpectedly the module correlated to plasma glucose levels in VAT contained primarily the same genes as the modules correlated to plasma HDL cholesterol levels in SAT. Moreover the genes differentially expressed in SAT and VAT were not correlated to any of the parameters we tested, and expression levels of the genes that were correlated to plasma HDL levels in SAT and glucose levels in VAT were similar in both tissue types. This could indicate that although gene expression levels in VAT and SAT are associated with different plasma parameters - glucose and HDL levels, respectively - the molecular perturbations that underlie these associations are the same. Further, it might imply that gene expression in SAT is a reasonably good "model" for gene expression in VAT in regard to HDL and glucose metabolism.

## Conclusions

In conclusion, our data confirm the genes and pathways that were associated to obesity in earlier studies; they are mainly related to immunity and metabolism and include immunity-related signalling pathways, the complement cascade, cholesterol metabolism and trafficking, lysosomal degradation and trafficking, and composition of the HDL particle. Our study also reveals large sets of novel genes differentially expressed across VAT and SAT and genes involved in HDL cholesterol and glucose metabolism parameters in obesity. Finally, we have shown that genes with expression levels correlated to plasma HDL cholesterol and glucose levels are the same in SAT and VAT, but whereas such genes correlate to plasma HDL levels in SAT, they correlate to plasma glucose levels in VAT.

## Competing interests

The authors declare that they have no competing interests.

## Authors' contributions

MW conceived the project, designed the study, performed microarray experiments, analyzed the microarray data, performed the quantitative Real Time PCR experiment, performed statistical analyses and drafted the manuscript. SR drafted the manuscript. EB performed the quantitative Real Time PCR experiment. FV collected patient samples. JG collected patient samples and designed the study. BS and MB performed microarray experiments. CW participated in design of the study and supervised all phases of the project including writing of the manuscript. TH drafted the manuscript. WB collected patient samples and designed the study. LF analyzed microarray data, designed the study and drafted the manuscript. MH designed the study, and supervised all phases of the project including writing of the manuscript.

All authors have read and approved the manuscript.

## Pre-publication history

The pre-publication history for this paper can be accessed here:

http://www.biomedcentral.com/1755-8794/3/34/prepub

## Supplementary Material

Additional file 1 **Table S1. Genes picked by random stratified selection and primer sequences used for qRT-PCR**. Overview and primer sequences of the genes selected through random stratified selection for the qRT-PCR validation experiment. Click here for file

Additional file 2 **Table S2. Correlations between age, BMI, and plasma parameters of the study population**. Overview of significant correlations between traits measured in the study population. Both Pearson correlation coefficients (left side of table) and p-values (right side of table) are shown for those correlations that have a p-value <0.01. Correlations significant after Bonferroni correction are marked with an asterisk. Data (except for age and BMI) have been transformed to a natural logarithm to obtain a normal distribution. TG, triglycerides; NEFA, non-esterified fatty acid; ALAT, alanine aminotransaminase; ASAT, aspartate aminotransaminase; CRP, C-reactive protein. Click here for file

Additional file 3 **Table S3. Overview of microarray and qRT-PCR results**. Comparison of qRT-PCR and microarray data from 20 randomly stratified selected genes in order to validate the quality of the microarray data. Click here for file

Additional file 4 **Figure S1. Correlation plot of microarray and qRT-PCR results**. Correlation plot of the fold changes in the microarray and the qRT-PCR experiments for 20 randomly stratified selected genes. The y-axis shows the Log2 averaged fold changes for the 20 genes tested as calculated in the qRT-PCR experiment. The x-axis shows Log2 averaged fold changes for these genes as detected in the microarray experiment. The fold changes obtained in the qRT-PCR and microarray experiments are strongly correlated (r = 0.88). Click here for file

Additional file 5 **Table S4A and S4B. Genes significantly upregulated in (A) subcutaneous and (B) visceral adipose tissue**. List of genes significantly upregulated in subcutaneous and visceral adipose tissue. Click here for file

Additional file 6 **Table S5A and S5B. Gene Set Enrichment Analysis of genes differentially expressed in (A) subcutaneous adipose tissue and (B) visceral adipose tissue**. Overview of biological processes overrepresented among genes differentially expressed in subcutaneous and visceral adipose tissue. Click here for file

Additional file 7 **Table S6A and S6B. Contents of the modules generated in (A) subcutaneous and (B) visceral adipose tissue**. For each module, the number of probes in the module, the number of genes corresponding to these probes, and the names of all those genes are listed. Click here for file

Additional file 8 **Figure S2. Coloured heatmap of modules in subcutaneous adipose tissue**. Pair-wise correlations between probes residing in all the modules identified in SAT were plotted. Probe pairs strongly positively correlated are shown in green and probe pairs strongly negatively correlated are shown in red. Colour intensity represents the strength of the correlation. The modules are indicated by white squares and are ordered in the same way as in Additional file 7, Tables S6A and S6B; thus with the largest module - containing the largest number of probes - in the upper left corner and the smallest module in the lower right corner. Click here for file

Additional file 9 **Figure S3. Coloured heatmap of modules in visceral adipose tissue**. Pair-wise correlations between probes residing in all the modules identified in VAT were plotted. Probe pairs strongly positively correlated are shown in green and probe pairs strongly negatively correlated are shown in red. Colour intensity represents the strength of the correlation. The modules are indicated by white squares and are ordered in the same way as in Additional file 7, Tables S6A and S6B; thus with the largest module - containing the largest number of probes - in the upper left corner and the smallest module in the lower right corner. Click here for file

Additional file 10 **Table S7A and S7B. Gene Set Enrichment Analysis of genes in the modules in (A) subcutaneous adipose tissue and (B) visceral adipose tissue**. Overview of biological processes overrepresented among modules identified in subcutaneous and visceral adipose tissue. Click here for file

Additional file 11 **Table S8. Relevant medication and menopausal status of the severely obese individuals**. Overview of prescribed medication relevant for tested traits, and menopausal status of the severely obese individuals. Patient ID's correspond with the patient ID's deposited in the Gene Expression Omnibus database. Patients that received no relevant medication are not included in the table. Dyslipidemia treatment consists of statins, and treatment of hypertension consists of ACE-inhibitors, Angiotensin Receptor Blockers, and Calcium antagonists. Click here for file

Additional file 12 **Table S9A and S9B. Correlations between modules in (A) subcutaneous adipose tissue and (B) visceral adipose tissue, and traits after correction for possible confounding factors**. Overview of correlations between modules and traits after correction for confounding factors - menopausal status, hormone treatment and treatment for diabetes, hypertension, dyslipidemia, along with all the other traits we measured: gender, age, BMI, plasma levels of glucose, insulin, HbA_1c_, triglycerides, non-esterified fatty acids, HDL cholesterol, LDL cholesterol, total cholesterol, CRP, ALAT, and ASAT - in (A) subcutaneous and (B) visceral adipose tissue. The last two columns in the table show Spearman rank correlation coefficients and p-values for the correlation between the module and the associated trait - as shown in columns 2 and 3 - corrected for potential confounders, which are listed in the first column. TG, triglycerides; NEFA, non-esterified fatty acid; ALAT, alanine aminotransaminase; ASAT, aspartate aminotransaminase; CRP, C-reactive protein. Click here for file

Additional file 13 **Table S10A, S10B, and S10C. Overlap in genes identified in our study and the study of Capel et al. in subcutaneous adipose tissue**. Overview of the overlap between the results of the present study and an earlier microarray study in subcutaneous adipose tissue of severely obese subjects performed by Capel et al. [11]. Click here for file
