# Supplementary material for: Co-expressed immune and metabolic genes in visceral and subcutaneous adipose tissue from severely obese individuals are associated with plasma HDL and glucose levels: a microarray study
Source: BMC Med Genomics. 2010 Aug 5;3:34. doi: 10.1186/1755-8794-3-34 (PMC2925326; doi:10.1186/1755-8794-3-34)
Supplement: Additional file 1 — Table S1. Genes picked by random stratified selection and primer sequences used for qRT-PCR. Overview and primer sequences of the genes selected through random stratified selection for the qRT-PCR validation experiment. [file 1755-8794-3-34-S1.DOC]

**Table S1. Genes picked by random stratified selection and primer sequences used for qRT-PCR.**

| Gene | Sequence forward primer | Sequence reverse primer |
| --- | --- | --- |
|  |  |  |
| *Upregulated in SAT* |  |  |
|  |  |  |
| SERPINA5 | TCTTTAAAGCTAAGTGGGAGAC | GTCCAGGAGGTAGTGATACTG |
| F2R | CAACTTCCTGCATTCATCTCAG | TGGGAGGCTGACTACAAACA |
| FMNL3 | GTAACTCGGAAGAAGTTCAGG | AGGTAATCCACCAGTACATCC |
| UGP2 | CACATGAATTTGAGCACACC | AGGGTTGAATCGAATCTTCAG |
| TRAK1 | GGCTTCTTGAGGAGAAAGAG | CCTCCTCCCTGATGTGTTC |
| NAV1 | CAACAATAGTGCTACGCACAG | CATCTTCAGGCTACCACTGTC |
| C18orf10 | AAGTGGGAAACCAGCATTAG | AGGCGGTAATAGGCAGTAAAG |
| COX4I2 | AAAGGTGGCCTTGTACCG | GCGAATCCAATGAAGAAGAAG |
| ZNF688 | GGAGAGGAGATGTCCCAAAC | TAATAGCTGGGTCAGGGTTG |
| CAP1 | CATTACACATGCCCTGAAAC | GTGCAGAGAATGGTTTGG |
|  |  |  |
| *Upregulated in VAT* |  |  |
|  |  |  |
| SGOL2 | GCAGTCTTTCTGAGTTCCATC | GAAGTTAATGGAACCCTTGC |
| IGFL2 | CTGGCTCCTGCTTATGTG | TGTAGATCTTGTCTCCACACC |
| REC8 | ATGGAGACTGAGCTACCC | CGAAAGGACTAGGAAGTCTG |
| TMEM1 | TGGAATGGAGAAGGTCCTATG | AAGGGAAACGTGAGCAGAG |
| SEMA4D | GAAGCAGCATGAGGTGTATTG | ACACACGTAAAGGGAAGTGG |
| SLC40A1 | TAGACTTAAAGTGGCCCAGAC | TCAGGATATAGCAGGAAGTGAG |
| PHGDH | AAGAAGTTCATGGGAACAGAG | GTCTTCATCCCAAAGGACTG |
| HDHD1A | GGAGAAGTGCCTTGTCTTTG | CAGATCTCGGCTCAAGTTTC |
| CLK1 | CTTAAGCACAATGTTAGAGG | CCAGACCATCTATTACCTTTC |
| KIAA0913 | AGAGTCGCATGGAGGTACTG | TAGCAGATCCTGGGCAAG |
|  |  |  |
| *House keeping gene* |  |  |
|  |  |  |
| TCEB2 | GGCAGATGACACCTTTGAG | TGTTCATTGGCACTGCTTC |
