# Supplementary material for: Co-expressed immune and metabolic genes in visceral and subcutaneous adipose tissue from severely obese individuals are associated with plasma HDL and glucose levels: a microarray study
Source: BMC Med Genomics. 2010 Aug 5;3:34. doi: 10.1186/1755-8794-3-34 (PMC2925326; doi:10.1186/1755-8794-3-34)
Supplement: Additional file 2 — Table S2. Correlations between age, BMI, and plasma parameters of the study population. Overview of significant correlations between traits measured in the study population. Both Pearson correlation coefficients (left side of table) and p-values (right side of table) are shown for those correlations that have a p-value <0.01. Correlations significant after Bonferroni correction are marked with an asterisk. Data (except for age and BMI) have been transformed to a natural logarithm to obtain a normal distribution. TG, triglycerides; NEFA, non-esterified fatty acid; ALAT, alanine aminotransaminase; ASAT, aspartate aminotransaminase; CRP, C-reactive protein. [file 1755-8794-3-34-S2.DOC]

**Table S2. Correlations between age, BMI, and plasma parameters of the study population.**

| P-value →  Pearson R ↓ |  |  |  |  |  |  |  |  |  |  |  |  |  |
| --- | --- | --- | --- | --- | --- | --- | --- | --- | --- | --- | --- | --- | --- |
|  | Age | BMI | Glucose | Insulin | HbA_1c_ | Total Cholesterol | HDL Cholesterol | LDL Cholesterol | TG | NEFA | ALAT | ASAT | CRP |
| Age |  |  |  |  |  |  |  |  |  |  |  |  |  |
| BMI |  |  |  | 5.3E-06* |  | 4.3E-03 | 6.0E-06* |  |  |  |  | 3.4E-03 | 1.2E-03 |
| Glucose |  |  |  |  | 2.1E-16* |  |  |  | 4.4E-07* |  |  |  |  |
| Insulin |  | 0.51* |  |  |  |  | 2.2E-05* |  |  |  |  |  |  |
| HbA_1c_ |  |  | 0.80* |  |  |  |  |  | 1.5E-07* |  | 1.3E-03 |  |  |
| Total Cholesterol |  | -0.34 |  |  |  |  |  | 1.1E-18* |  |  |  |  |  |
| HDL Cholesterol |  | -0.52* |  | -0.48* |  |  |  |  | 2.4E-03 |  |  |  | 7.8E-03 |
| LDL Cholesterol |  |  |  |  |  | 0.83* |  |  |  |  |  |  |  |
| TG |  |  | 0.56* |  | 0.58* |  | -0.36 |  |  | 3.4E-03 |  |  |  |
| NEFA |  |  |  |  |  |  |  |  | 0.36 |  |  | 4.9E-03 |  |
| ALAT |  |  |  |  | 0.38 |  |  |  |  |  |  | 4.1E-05* |  |
| ASAT |  | 0.35 |  |  |  |  |  |  |  | 0.35 | 0.47* |  |  |
| CRP |  | 0.39 |  |  |  |  | -0.32 |  |  |  |  |  |  |
