# Supplementary material for: Co-expressed immune and metabolic genes in visceral and subcutaneous adipose tissue from severely obese individuals are associated with plasma HDL and glucose levels: a microarray study
Source: BMC Med Genomics. 2010 Aug 5;3:34. doi: 10.1186/1755-8794-3-34 (PMC2925326; doi:10.1186/1755-8794-3-34)
Supplement: Additional file 3 — Table S3. Overview of microarray and qRT-PCR results. Comparison of qRT-PCR and microarray data from 20 randomly stratified selected genes in order to validate the quality of the microarray data. [file 1755-8794-3-34-S3.DOC]

**Table S3. Overview of microarray and qRT-PCR results.**

| Gene | Probe ID | P-value microarray | Fold change microarray | Fold change qRT-PCR |
| --- | --- | --- | --- | --- |
|  |  |  |  |  |
| *Upregulated in SAT* | | | | |
| SERPINA5 | 3290630 | 0.00902344 | 19.644186 | 10.16856 |
| F2R | 4570398 | 0.00902344 | 2.64809 | 1.609912 |
| FMNL3 | 150551 | 0.00902344 | 2.4015281 | 1.404747 |
| UGP2 | 3840053 | 0.047201768 | 1.8387715 | 1.473953 |
| TRAK1 | 6270347 | 0.028280124 | 1.8087976 | 1.164031 |
| NAV1 | 940685 | 0.00902344 | 1.5992578 | 1.432248 |
| C18orf10 | 2690541 | 0.047201768 | 1.5522914 | 1.338361 |
| COX4I2 | 3360333 | 0.047201768 | 1.376744 | 1.304104 |
| ZNF688 | 3190048 | 0.028280124 | 1.3380661 | 1.539814 |
| CAP1 | 1070754 | 0.0162936 | 1.1830256 | 1.209079 |
|  |  |  |  |  |
| *Upregulated in VAT* | | | | |
| SGOL2 | 3520754 | 0.00902344 | 30.382286 | 6.340709 |
| IGFL2 | 4730523 | 0.018602934 | 19.863186 | 9.137275 |
| REC8 | 4830338 | 0.0162936 | 5.4568076 | 4.746296 |
| TMEM1 | 5910082 | 0.047201768 | 4.64141 | 1.048041 |
| SEMA4D | 4880408 | 0.00902344 | 2.550226 | 2.450011 |
| SLC40A1 | 730164 | 0.00902344 | 2.146016 | 2.372214 |
| PHGDH | 240086 | 0.047201768 | 1.685529 | 1.958099 |
| HDHD1A | 130228 | 0.028280124 | 1.5792596 | 1.359239 |
| CLK1 | 3440138 | 0.047201768 | 1.3790543 | 0.900313 |
| KIAA0913 | 5890136 | 0.028280124 | 1.1733673 | 1.359315 |
