# Supplementary material for: Co-expressed immune and metabolic genes in visceral and subcutaneous adipose tissue from severely obese individuals are associated with plasma HDL and glucose levels: a microarray study
Source: BMC Med Genomics. 2010 Aug 5;3:34. doi: 10.1186/1755-8794-3-34 (PMC2925326; doi:10.1186/1755-8794-3-34)
Supplement: Additional file 4 — Figure S1. Correlation plot of microarray and qRT-PCR results. Correlation plot of the fold changes in the microarray and the qRT-PCR experiments for 20 randomly stratified selected genes. The y-axis shows the Log2 averaged fold changes for the 20 genes tested as calculated in the qRT-PCR experiment. The x-axis shows Log2 averaged fold changes for these genes as detected in the microarray experiment. The fold changes obtained in the qRT-PCR and microarray experiments are strongly correlated (r = 0.88). [file 1755-8794-3-34-S4.DOC]

**Figure S1. Correlation plot of microarray and qRT-PCR results.**
