# Supplementary figures and images for: Co-expressed immune and metabolic genes in visceral and subcutaneous adipose tissue from severely obese individuals are associated with plasma HDL and glucose levels: a microarray study
Source: BMC Med Genomics. 2010 Aug 5;3:34. doi: 10.1186/1755-8794-3-34 (PMC2925326; doi:10.1186/1755-8794-3-34)

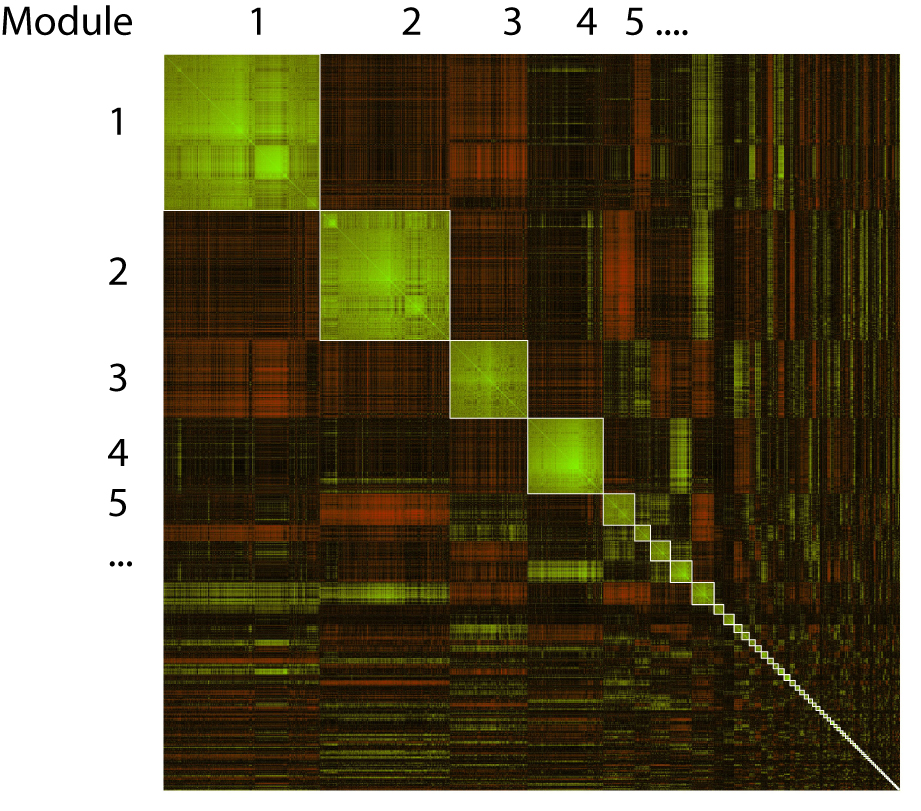

Supplement: Additional file 8 — Figure S2. Coloured heatmap of modules in subcutaneous adipose tissue. Pair-wise correlations between probes residing in all the modules identified in SAT were plotted. Probe pairs strongly positively correlated are shown in green and probe pairs strongly negatively correlated are shown in red. Colour intensity represents the strength of the correlation. The modules are indicated by white squares and are ordered in the same way as in Additional file 7, Tables S6A and S6B; thus with the largest module - containing the largest number of probes - in the upper left corner and the smallest module in the lower right corner. [file 1755-8794-3-34-S8.JPEG]

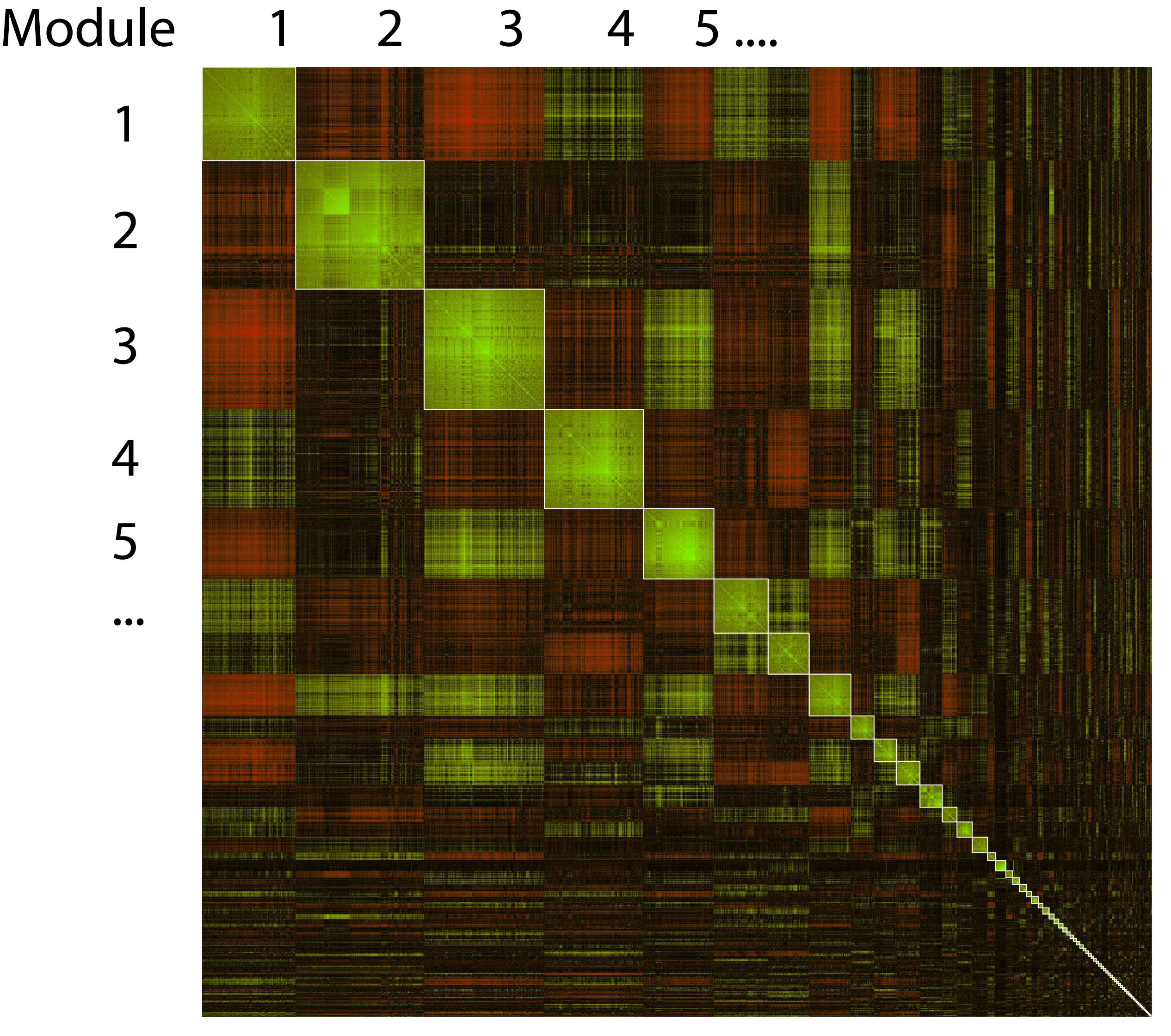

Supplement: Additional file 9 — Figure S3. Coloured heatmap of modules in visceral adipose tissue. Pair-wise correlations between probes residing in all the modules identified in VAT were plotted. Probe pairs strongly positively correlated are shown in green and probe pairs strongly negatively correlated are shown in red. Colour intensity represents the strength of the correlation. The modules are indicated by white squares and are ordered in the same way as in Additional file 7, Tables S6A and S6B; thus with the largest module - containing the largest number of probes - in the upper left corner and the smallest module in the lower right corner. [file 1755-8794-3-34-S9.JPEG]
