# Supplementary material for: Co-expressed immune and metabolic genes in visceral and subcutaneous adipose tissue from severely obese individuals are associated with plasma HDL and glucose levels: a microarray study
Source: BMC Med Genomics. 2010 Aug 5;3:34. doi: 10.1186/1755-8794-3-34 (PMC2925326; doi:10.1186/1755-8794-3-34)
Supplement: Additional file 11 — Table S8. Relevant medication and menopausal status of the severely obese individuals. Overview of prescribed medication relevant for tested traits, and menopausal status of the severely obese individuals. Patient ID's correspond with the patient ID's deposited in the Gene Expression Omnibus database. Patients that received no relevant medication are not included in the table. Dyslipidemia treatment consists of statins, and treatment of hypertension consists of ACE-inhibitors, Angiotensin Receptor Blockers, and Calcium antagonists. [file 1755-8794-3-34-S11.DOC]

**Table S8. Relevant medication and menopausal status of the severely obese individuals.**

| Patient ID | Menopause | Medication | | | | | | |
| --- | --- | --- | --- | --- | --- | --- | --- | --- |
|  |  | Microgynon | Insulin | Metformin | Pioglitazone | Sulfonylureas | Dyslipidemia treatment | Treatment of hypertension |
| 36 |  |  | X |  |  |  | X |  |
| 37 |  |  |  | X |  |  |  |  |
| 40 | X |  |  |  |  |  |  | X |
| 43 |  |  | X | X |  |  |  |  |
| 51 | X |  | X |  |  |  | X |  |
| 55 |  |  | X | X |  |  | X |  |
| 56 |  |  |  |  |  |  | X |  |
| 56 |  |  |  |  |  |  |  | X |
| 57 |  |  |  | X |  |  |  | X |
| 59 |  |  |  |  |  |  |  | X |
| 60 |  |  |  | X |  |  |  |  |
| 63 | X |  |  |  |  |  |  |  |
| 65 |  |  |  |  |  |  | X |  |
| 69 |  |  |  | X |  |  |  |  |
| 75 |  |  |  | X |  |  |  | X |
| 77 |  |  |  |  |  |  | X |  |
| 78 |  |  | X | X |  |  | X |  |
| 85 | X |  |  | X |  |  |  |  |
| 87 |  |  |  | X |  |  | X | X |
| 95 | X |  |  |  |  |  |  | X |
| 96 | X |  |  |  |  |  |  |  |
| 97 | X |  |  |  |  |  |  |  |
| 99 |  |  |  |  |  |  | X | X |
| 101 |  |  | X |  | X |  | X | X |
| 102 |  |  |  |  |  |  |  | X |
| 104 | X |  |  |  |  |  |  |  |
| 105 |  |  |  |  |  |  | X | X |
| 107 | X |  |  |  |  |  |  | X |
| 109 | X |  |  |  |  |  | X | X |
| 110 |  | X |  |  |  |  |  |  |
| 111 |  | X |  |  |  |  | X |  |
| 112 |  |  |  |  |  |  | X |  |
| 114 |  | X |  |  |  |  |  |  |
| 115 | X |  |  |  |  |  |  |  |
| Total number  of patients | 11 | 3 | 6 | 10 | 1 | 0 | 14 | 13 |
