# Supplementary material for: Co-expressed immune and metabolic genes in visceral and subcutaneous adipose tissue from severely obese individuals are associated with plasma HDL and glucose levels: a microarray study
Source: BMC Med Genomics. 2010 Aug 5;3:34. doi: 10.1186/1755-8794-3-34 (PMC2925326; doi:10.1186/1755-8794-3-34)
Supplement: Additional file 12 — Table S9A and S9B. Correlations between modules in (A) subcutaneous adipose tissue and (B) visceral adipose tissue, and traits after correction for possible confounding factors. Overview of correlations between modules and traits after correction for confounding factors - menopausal status, hormone treatment and treatment for diabetes, hypertension, dyslipidemia, along with all the other traits we measured: gender, age, BMI, plasma levels of glucose, insulin, HbA1c, triglycerides, non-esterified fatty acids, HDL cholesterol, LDL cholesterol, total cholesterol, CRP, ALAT, and ASAT - in (A) subcutaneous and (B) visceral adipose tissue. The last two columns in the table show Spearman rank correlation coefficients and p-values for the correlation between the module and the associated trait - as shown in columns 2 and 3 - corrected for potential confounders, which are listed in the first column. TG, triglycerides; NEFA, non-esterified fatty acid; ALAT, alanine aminotransaminase; ASAT, aspartate aminotransaminase; CRP, C-reactive protein. [file 1755-8794-3-34-S12.DOC]

**Table S9A. Correlations between modules in subcutaneous adipose tissue and traits after correction for possible confounding factors.**

| **Covariate** | **Module ID** | **Trait** | **Correlation coefficient** | **P-value** |
| --- | --- | --- | --- | --- |
|  |  |  |  |  |
| Dyslipidemia treatment | SAT 31 | Gender | 0.75 | 2.26E-14 |
| Hormone therapy | SAT 31 | Gender | 0.74 | 8.70E-14 |
| Hypertension treatment | SAT 31 | Gender | 0.74 | 1.21E-13 |
| Menopause | SAT 31 | Gender | 0.72 | 9.34E-13 |
| Glucose | SAT 31 | Gender | 0.71 | 2.38E-12 |
| NEFA | SAT 31 | Gender | 0.71 | 7.59E-11 |
| Diabetes treatment | SAT 31 | Gender | 0.67 | 1.29E-10 |
| ASAT | SAT 31 | Gender | 0.67 | 5.81E-10 |
| HbA_1c_ | SAT 31 | Gender | 0.66 | 1.04E-09 |
| TG | SAT 31 | Gender | 0.65 | 1.52E-09 |
| Insulin | SAT 31 | Gender | 0.64 | 2.54E-09 |
| HDL cholesterol | SAT 31 | Gender | 0.64 | 4.42E-09 |
| Total cholesterol | SAT 31 | Gender | 0.64 | 6.21E-09 |
| LDL cholesterol | SAT 31 | Gender | 0.62 | 1.30E-08 |
| BMI | SAT 31 | Gender | 0.61 | 1.39E-08 |
| CRP | SAT 31 | Gender | 0.62 | 2.05E-08 |
| Age | SAT 31 | Gender | 0.56 | 2.72E-07 |
| ALAT | SAT 31 | Gender | 0.56 | 6.23E-07 |
| Gender | SAT 31 | Gender | 0.00 | 1 |
|  |  |  |  |  |
| Age | SAT 4 | HDL | -0.63 | 7.25E-09 |
| Hypertension treatment | SAT 4 | HDL | -0.60 | 8.47E-08 |
| Dyslipidemia treatment | SAT 4 | HDL | -0.58 | 2.66E-07 |
| Hormone therapy | SAT 4 | HDL | -0.58 | 2.76E-07 |
| ALAT | SAT 4 | HDL | -0.57 | 4.73E-07 |
| Gender | SAT 4 | HDL | -0.56 | 7.15E-07 |
| LDL cholesterol | SAT 4 | HDL | -0.56 | 7.72E-07 |
| CRP | SAT 4 | HDL | -0.55 | 1.41E-06 |
| Diabetes treatment | SAT 4 | HDL | -0.54 | 2.14E-06 |
| ASAT | SAT 4 | HDL | -0.54 | 2.37E-06 |
| NEFA | SAT 4 | HDL | -0.55 | 2.45E-06 |
| HbA_1c_ | SAT 4 | HDL | -0.54 | 2.74E-06 |
| Menopause | SAT 4 | HDL | -0.53 | 3.21E-06 |
| Glucose | SAT 4 | HDL | -0.53 | 3.92E-06 |
| BMI | SAT 4 | HDL | -0.53 | 4.11E-06 |
| Insulin | SAT 4 | HDL | -0.52 | 6.78E-06 |
| Total cholesterol | SAT 4 | HDL | -0.51 | 1.04E-05 |
| TG | SAT 4 | HDL | -0.50 | 1.53E-05 |
| HDL cholesterol | SAT 4 | HDL | 0.00 | 1 |
|  |  |  |  |  |
| Menopause | SAT 13 | Glucose | -0.53 | 2.19E-06 |
| Insulin | SAT 13 | Glucose | -0.53 | 2.24E-06 |
| Gender | SAT 13 | Glucose | -0.52 | 3.21E-06 |
| LDL cholesterol | SAT 13 | Glucose | -0.53 | 3.44E-06 |
| Total cholesterol | SAT 13 | Glucose | -0.53 | 3.96E-06 |
| CRP | SAT 13 | Glucose | -0.53 | 5.06E-06 |
| Hormone therapy | SAT 13 | Glucose | -0.51 | 5.56E-06 |
| Hypertension treatment | SAT 13 | Glucose | -0.49 | 1.16E-05 |
| Age | SAT 13 | Glucose | -0.46 | 3.87E-05 |
| BMI | SAT 13 | Glucose | -0.46 | 4.62E-05 |
| Dyslipidemia treatment | SAT 13 | Glucose | -0.45 | 8.09E-05 |
| NEFA | SAT 13 | Glucose | -0.44 | 3.13E-04 |
| Diabetes treatment | SAT 13 | Glucose | -0.41 | 3.18E-04 |
| HDL cholesterol | SAT 13 | Glucose | -0.40 | 7.36E-04 |
| ALAT | SAT 13 | Glucose | -0.37 | 0.00191 |
| ASAT | SAT 13 | Glucose | -0.34 | 0.00426 |
| HbA_1c_ | SAT 13 | Glucose | -0.23 | 0.0553 |
| TG | SAT 13 | Glucose | -0.14 | 0.255 |
| Glucose | SAT 13 | Glucose | 0.00 | 1 |
|  |  |  |  |  |
| Menopause | SAT 13 | TG | -0.56 | 7.24E-07 |
| Gender | SAT 13 | TG | -0.53 | 2.94E-06 |
| Total cholesterol | SAT 13 | TG | -0.53 | 3.99E-06 |
| BMI | SAT 13 | TG | -0.51 | 9.03E-06 |
| Hypertension treatment | SAT 13 | TG | -0.48 | 3.51E-05 |
| Hormone therapy | SAT 13 | TG | -0.48 | 3.64E-05 |
| Insulin | SAT 13 | TG | -0.48 | 3.64E-05 |
| LDL cholesterol | SAT 13 | TG | -0.47 | 4.43E-05 |
| CRP | SAT 13 | TG | -0.48 | 5.04E-05 |
| Age | SAT 13 | TG | -0.47 | 5.06E-05 |
| Dyslipidemia treatment | SAT 13 | TG | -0.41 | 4.41E-04 |
| ALAT | SAT 13 | TG | -0.42 | 4.51E-04 |
| Diabetes treatment | SAT 13 | TG | -0.39 | 0.00105 |
| NEFA | SAT 13 | TG | -0.37 | 0.00249 |
| ASAT | SAT 13 | TG | -0.36 | 0.00261 |
| HDL cholesterol | SAT 13 | TG | -0.33 | 0.00557 |
| HbA_1c_ | SAT 13 | TG | -0.20 | 0.108 |
| Glucose | SAT 13 | TG | -0.10 | 0.406 |
| TG | SAT 13 | TG | 0.00 | 1 |
|  |  |  |  |  |
| Age | SAT 8 | HDL | -0.55 | 1.25E-06 |
| Gender | SAT 8 | HDL | -0.50 | 1.33E-05 |
| Hypertension treatment | SAT 8 | HDL | -0.50 | 1.71E-05 |
| ALAT | SAT 8 | HDL | -0.49 | 2.74E-05 |
| Dyslipidemia treatment | SAT 8 | HDL | -0.48 | 2.98E-05 |
| LDL cholesterol | SAT 8 | HDL | -0.47 | 4.70E-05 |
| Total cholesterol | SAT 8 | HDL | -0.47 | 5.03E-05 |
| ASAT | SAT 8 | HDL | -0.47 | 5.32E-05 |
| Hormone therapy | SAT 8 | HDL | -0.47 | 5.65E-05 |
| Glucose | SAT 8 | HDL | -0.46 | 9.65E-05 |
| HbA_1c_ | SAT 8 | HDL | -0.45 | 1.14E-04 |
| NEFA | SAT 8 | HDL | -0.46 | 1.52E-04 |
| Diabetes treatment | SAT 8 | HDL | -0.43 | 2.22E-04 |
| CRP | SAT 8 | HDL | -0.43 | 3.43E-04 |
| TG | SAT 8 | HDL | -0.42 | 3.78E-04 |
| Menopause | SAT 8 | HDL | -0.41 | 4.50E-04 |
| Insulin | SAT 8 | HDL | -0.37 | 0.00193 |
| BMI | SAT 8 | HDL | -0.37 | 0.00203 |
| HDL cholesterol | SAT 8 | HDL | 0.00 | 1 |
|  |  |  |  |  |
| Age | SAT 39 | HDL | -0.58 | 1.86E-07 |
| Hormone therapy | SAT 39 | HDL | -0.50 | 1.35E-05 |
| ASAT | SAT 39 | HDL | -0.50 | 1.45E-05 |
| Hypertension treatment | SAT 39 | HDL | -0.49 | 2.58E-05 |
| Menopause | SAT 39 | HDL | -0.49 | 2.72E-05 |
| Dyslipidemia treatment | SAT 39 | HDL | -0.48 | 2.96E-05 |
| ALAT | SAT 39 | HDL | -0.47 | 4.90E-05 |
| Total cholesterol | SAT 39 | HDL | -0.47 | 6.09E-05 |
| Gender | SAT 39 | HDL | -0.46 | 6.82E-05 |
| LDL cholesterol | SAT 39 | HDL | -0.46 | 7.73E-05 |
| NEFA | SAT 39 | HDL | -0.47 | 8.60E-05 |
| BMI | SAT 39 | HDL | -0.45 | 1.37E-04 |
| HbA_1c_ | SAT 39 | HDL | -0.45 | 1.57E-04 |
| Diabetes treatment | SAT 39 | HDL | -0.44 | 1.63E-04 |
| Glucose | SAT 39 | HDL | -0.43 | 2.38E-04 |
| CRP | SAT 39 | HDL | -0.44 | 2.58E-04 |
| TG | SAT 39 | HDL | -0.41 | 4.71E-04 |
| Insulin | SAT 39 | HDL | -0.40 | 6.21E-04 |
| HDL cholesterol | SAT 39 | HDL | 0.00 | 1 |

**Table S9B. Correlations between modules in visceral adipose tissue and traits after correction for possible confounding factors.**

| **Covariate** | **Module ID** | **Trait** | **Correlation coefficient** | **P-value** |
| --- | --- | --- | --- | --- |
|  |  |  |  |  |
| Diabetes treatment | VAT 31 | Gender | 0.77 | 7.06E-15 |
| Menopause | VAT 31 | Gender | 0.77 | 1.78E-14 |
| Hormone therapy | VAT 31 | Gender | 0.77 | 1.95E-14 |
| Dyslipidemia treatment | VAT 31 | Gender | 0.75 | 8.46E-14 |
| ALAT | VAT 31 | Gender | 0.75 | 9.75E-13 |
| Hypertension treatment | VAT 31 | Gender | 0.70 | 1.66E-11 |
| NEFA | VAT 31 | Gender | 0.73 | 1.74E-11 |
| HDL cholesterol | VAT 31 | Gender | 0.71 | 3.99E-11 |
| Glucose | VAT 31 | Gender | 0.67 | 3.87E-10 |
| Total cholesterol | VAT 31 | Gender | 0.66 | 4.39E-09 |
| HbA_1c_ | VAT 31 | Gender | 0.65 | 7.22E-09 |
| CRP | VAT 31 | Gender | 0.64 | 1.07E-08 |
| LDL cholesterol | VAT 31 | Gender | 0.64 | 1.59E-08 |
| Age | VAT 31 | Gender | 0.60 | 6.22E-08 |
| ASAT | VAT 31 | Gender | 0.61 | 7.41E-08 |
| Insulin | VAT 31 | Gender | 0.61 | 7.55E-08 |
| BMI | VAT 31 | Gender | 0.59 | 1.38E-07 |
| TG | VAT 31 | Gender | 0.60 | 1.84E-07 |
| Gender | VAT 31 | Gender | 0.00 | 1 |
|  |  |  |  |  |
| LDL cholesterol | VAT 40 | Insulin | -0.58 | 5.46E-07 |
| Diabetes treatment | VAT 40 | Insulin | -0.57 | 6.90E-07 |
| HbA_1c_ | VAT 40 | Insulin | -0.58 | 7.75E-07 |
| Total cholesterol | VAT 40 | Insulin | -0.57 | 8.41E-07 |
| CRP | VAT 40 | Insulin | -0.57 | 1.03E-06 |
| TG | VAT 40 | Insulin | -0.56 | 1.75E-06 |
| Age | VAT 40 | Insulin | -0.55 | 1.99E-06 |
| Glucose | VAT 40 | Insulin | -0.55 | 2.58E-06 |
| ALAT | VAT 40 | Insulin | -0.55 | 3.61E-06 |
| Dyslipidemia treatment | VAT 40 | Insulin | -0.53 | 4.97E-06 |
| Menopause | VAT 40 | Insulin | -0.53 | 6.03E-06 |
| NEFA | VAT 40 | Insulin | -0.53 | 1.20E-05 |
| ASAT | VAT 40 | Insulin | -0.51 | 2.17E-05 |
| Hormone therapy | VAT 40 | Insulin | -0.49 | 3.20E-05 |
| Hypertension treatment | VAT 40 | Insulin | -0.46 | 1.09E-04 |
| HDL cholesterol | VAT 40 | Insulin | -0.45 | 2.03E-04 |
| Gender | VAT 40 | Insulin | -0.42 | 4.89E-04 |
| BMI | VAT 40 | Insulin | -0.34 | 0.00566 |
| Insulin | VAT 40 | Insulin | 0.00 | 1 |
|  |  |  |  |  |
| HbA_1c_ | VAT 40 | BMI | -0.52 | 1.03E-05 |
| Age | VAT 40 | BMI | -0.50 | 1.15E-05 |
| Dyslipidemia treatment | VAT 40 | BMI | -0.49 | 1.93E-05 |
| TG | VAT 40 | BMI | -0.51 | 1.95E-05 |
| Menopause | VAT 40 | BMI | -0.49 | 2.07E-05 |
| Hypertension treatment | VAT 40 | BMI | -0.49 | 2.38E-05 |
| Diabetes treatment | VAT 40 | BMI | -0.48 | 2.84E-05 |
| Glucose | VAT 40 | BMI | -0.48 | 3.33E-05 |
| Total cholesterol | VAT 40 | BMI | -0.49 | 6.00E-05 |
| LDL cholesterol | VAT 40 | BMI | -0.48 | 6.61E-05 |
| CRP | VAT 40 | BMI | -0.47 | 1.21E-04 |
| ALAT | VAT 40 | BMI | -0.45 | 2.01E-04 |
| NEFA | VAT 40 | BMI | -0.45 | 3.21E-04 |
| Hormone therapy | VAT 40 | BMI | -0.42 | 4.24E-04 |
| Gender | VAT 40 | BMI | -0.40 | 7.88E-04 |
| ASAT | VAT 40 | BMI | -0.40 | 0.00115 |
| HDL cholesterol | VAT 40 | BMI | -0.35 | 0.00475 |
| Insulin | VAT 40 | BMI | -0.25 | 0.0469 |
| BMI | VAT 40 | BMI | 0.00 | 1 |
|  |  |  |  |  |
| Gender | VAT 9 | Glucose | 0.50 | 1.34E-05 |
| Hormone therapy | VAT 9 | Glucose | 0.49 | 1.80E-05 |
| Hypertension treatment | VAT 9 | Glucose | 0.49 | 2.06E-05 |
| Insulin | VAT 9 | Glucose | 0.49 | 3.29E-05 |
| Menopause | VAT 9 | Glucose | 0.47 | 5.08E-05 |
| BMI | VAT 9 | Glucose | 0.47 | 5.70E-05 |
| Diabetes treatment | VAT 9 | Glucose | 0.45 | 1.04E-04 |
| Dyslipidemia treatment | VAT 9 | Glucose | 0.43 | 2.99E-04 |
| Total cholesterol | VAT 9 | Glucose | 0.44 | 3.38E-04 |
| ALAT | VAT 9 | Glucose | 0.43 | 3.85E-04 |
| LDL cholesterol | VAT 9 | Glucose | 0.42 | 5.10E-04 |
| Age | VAT 9 | Glucose | 0.40 | 7.19E-04 |
| NEFA | VAT 9 | Glucose | 0.40 | 0.00139 |
| HDL cholesterol | VAT 9 | Glucose | 0.37 | 0.00279 |
| ASAT | VAT 9 | Glucose | 0.37 | 0.00288 |
| CRP | VAT 9 | Glucose | 0.35 | 0.00514 |
| HbA_1c_ | VAT 9 | Glucose | 0.28 | 0.0276 |
| TG | VAT 9 | Glucose | 0.27 | 0.0310 |
| Glucose | VAT 9 | Glucose | 0.00 | 1 |
