# Supplementary material for: Co-expressed immune and metabolic genes in visceral and subcutaneous adipose tissue from severely obese individuals are associated with plasma HDL and glucose levels: a microarray study
Source: BMC Med Genomics. 2010 Aug 5;3:34. doi: 10.1186/1755-8794-3-34 (PMC2925326; doi:10.1186/1755-8794-3-34)
Supplement: Additional file 13 — Table S10A, S10B, and S10C. Overlap in genes identified in our study and the study of Capel et al. in subcutaneous adipose tissue. Overview of the overlap between the results of the present study and an earlier microarray study in subcutaneous adipose tissue of severely obese subjects performed by Capel et al. [11]. [file 1755-8794-3-34-S13.DOC]

**Table S10A, S10B, and S10C. Overlap in genes identified in our study and the study of Capel et al. in subcutaneous adipose tissue.**

Capel et al [11] performed a genome-wide expression study in morbidly obese individuals during weight loss induced by a dietary intervention. This dietary intervention consisted of two phases: 1) stringent caloric restriction and weight loss and 2) less stringent caloric restriction and weight maintenance. Subcutaneous adipose tissue was sampled at baseline, after stringent caloric restriction, and at the end of the weight maintenance phase. Genome-wide expression levels were compared between baseline and after stringent diet (Energy Restriction phase; ER), stringent diet and maintenance diet (Weight Stabilization phase; WS), and baseline and maintenance diet (whole Dietary Intervention; DI). In addition 31 genes specifically expressed in macrophages were analyzed using quantitative RT-PCR.

A.

| Capel et al →    Present study ↓ |  | Energy restriction | | Weight stabilization | | Dietary intervention | |
| --- | --- | --- | --- | --- | --- | --- | --- |
|  |  | Down | Up | Down | Up | Down | Up |
|  | Total | 500 | 40 | 535 | 235 | 461 | 87 |
|  |  |  |  |  |  |  |  |
| SAT Module 4 | 308 | 0 | 1 | 97 | 0 | 77 | 0 |
| SAT Module 8 | 87 | 1 | 0 | 37 | 0 | 39 | 0 |
| SAT Module 13 | 28 | 0 | 4 | 2 | 2 | 0 | 5 |
| SAT Module 39 | 7 | 1 | 0 | 0 | 0 | 0 | 0 |
|  |  |  |  |  |  |  |  |
| VAT module 9 | 103 | 1 | 0 | 42 | 0 | 41 | 0 |
| VAT module 40 | 12 | 0 | 0 | 0 | 2 | 0 | 0 |

Table S10A shows the overlap between the genes in the modules correlated to a plasma parameter identified in the present study, and the genes up- or downregulated during different stages of dietary intervention in the whole SAT in the study of Capel (according to [11] supplemental table 1). Modules SAT 4, SAT 8, and VAT 9 found in the present study contain a significant amount of genes that were found by Capel to be downregulated during the weight stabilization phase and the whole dietary intervention. Module SAT 13 contains genes upregulated during the energy restriction phase and the whole dietary intervention.

B.

| Capel et al →    Present study ↓ |  | Adipocyte genes differently expressed DI | Macrophage genes differently expressed DI |
| --- | --- | --- | --- |
|  |  |  |  |
|  | Total | 82 | 58 |
|  |  |  |  |
| SAT Module 4 | 308 | 0 | 13 |
| SAT Module 8 | 87 | 0 | 15 |
| SAT Module 13 | 28 | 0 | 0 |
| SAT Module 39 | 7 | 0 | 0 |
|  |  |  |  |
| VAT module 9 | 103 | 0 | 15 |
| VAT module 40 | 12 | 0 | 0 |

Table S10B shows the overlap between the genes in the modules correlated to a plasma parameter identified in the present study, and the genes differentially expressed during the whole dietary intervention in relation to metabolism and macrophage activity in the study of Capel (according to [11] supplemental tables A6 and A7). Modules SAT 4, SAT 8, and VAT 9 found in the present study contain a significant amount of genes that were found by Capel to have different expression after weight loss in the set of genes related to macrophage activity. There is no overlap between genes in any of the modules correlated to a metabolic trait in the present study and genes differentially expressed during whole dietary intervention in the set of genes related to metabolism in the study of Capel.

C.

| Capel et al →    Present study ↓ |  | Predictor of INS sensitivity ER | Predictor of INS sensitivity WS | Predictor of INS sensitivity DI |
| --- | --- | --- | --- | --- |
|  |  |  |  |  |
|  | Total | 76 | 101 | 100 |
|  |  |  |  |  |
| SAT Module 4 | 308 | 15 | 25 | 39 |
| SAT Module 8 | 87 | 1 | 7 | 15 |
| SAT Module 13 | 28 | 4 | 0 | 2 |
| SAT Module 39 | 7 | 0 | 0 | 1 |
|  |  |  |  |  |
| VAT module 9 | 103 | 1 | 9 | 17 |
| VAT module 40 | 12 | 0 | 0 | 0 |

Table S10C shows that modules SAT 4, SAT 8, SAT 13 and VAT 9 in the present study, contain a significant amount of genes that were identified in the study of Capel to be predictors of insulin sensitivity in regard to gene expression during all the phases of the dietary intervention (according to [11] supplemental table A5).
